# Antiphospholipid Syndrome Nephropathy: From Pathogenesis to Treatment

**DOI:** 10.3389/fimmu.2018.01181

**Published:** 2018-05-31

**Authors:** Maria G. Tektonidou

**Affiliations:** Joint Rheumatology Academic Program, First Department of Propaedeutic Internal Medicine, School of Medicine, National and Kapodistrian University of Athens, Athens, Greece

**Keywords:** antiphospholipid antibodies, antiphospholipid syndrome, nephropathy, pathogenesis, treatment

## Abstract

Kidney damage is a well-recognized complication of the antiphospholipid syndrome (APS), either primary or systemic lupus erythematosus (SLE)-associated APS. Kidney involvement in APS involves a variety of manifestations, such as renal artery thrombosis or stenosis, renal vein thrombosis, allograft loss due to thrombosis after kidney transplantation, and injury to the renal microvasculature, also known as APS nephropathy. Biopsy in patients with APS nephropathy includes acute thrombotic microangiopathy lesions and chronic intrarenal vascular lesions such as interlobular fibrous intimal hyperplasia, arterial and arteriolar recanalizing thrombosis, fibrous arterial occlusion, and focal cortical atrophy. The most frequent clinical features are hypertension, microscopic hematuria, proteinuria (ranging from mild to nephritic levels), and renal insufficiency. It is uncertain whether antiphospholipid antibodies or other factors are implicated in the development of APS nephropathy, and whether it is driven mainly by thrombotic or by inflammatory processes. Experimental models and clinical studies of thrombotic microangiopathy lesions implicate activation of the complement cascade, tissue factor, and the mTORC pathway. Currently, the management of APS nephropathy relies on expert opinion, and consensus is lacking. There is limited evidence about the effect of anticoagulants, and their use remains controversial. Treatment approaches in patients with APS nephropathy lesions may include the use of heparin based on its role on complement activation pathway inhibition or the use of intravenous immunoglobulin and/or plasma exchange. Targeted therapies may also be considered based on potential APS nephropathy pathogenetic mechanisms such as B-cell directed therapies, complement inhibition, tissue factor inhibition, mTOR pathway inhibition, or anti-interferon antibodies, but prospective multicenter studies are needed to address their role.

## Introduction

Antiphospholipid syndrome (APS) is a systemic autoimmune disorder characterized by thrombotic episodes in the arterial or venous circulation, in the presence of antiphospholipid antibodies (aPL), namely lupus anticoagulant (LA), anticardiolipin antibodies, and anti-β2glycoprotein-I antibodies (anti-β2GPI) (1). APS can be either primary or secondary when it occurs in the context of other underlying autoimmune disorder, mainly systemic lupus erythematosus (SLE). aPL positivity may occur in 0–5% of healthy individuals and in approximately 30–40% of patients with SLE; one third of SLE patients with positive aPL develop thrombosis during their follow-up.

Antiphospholipid syndrome can affect any part of kidney vasculature such as renal arteries and veins, intrarenal arteries and arterioles, and glomerular capillaries (2). In addition to thrombotic manifestations from the large renal vessels that are part of the updated Sapporo criteria for APS, characteristic microvascular nephropathy lesions are included in the non-criteria manifestations of APS (1, 2).

## Lupus Nephritis and aPL

A number of studies have shown that aPL positivity is a poor prognostic factor in lupus nephritis (3, 4). In a study of 111 SLE patients with nephritis and a mean follow-up of 14 years, the presence of positive aPL (*p* = 0.02) was identified as independent predictor of chronic renal function deterioration (5). Natejumnong et al. showed that patients with SLE nephritis and LA positivity had higher systolic blood pressure (133.7 versus 121.9 mmHg, *p* = 0.005), serum creatinine (233.0 versus 94.9 μmol/L), and 24-h urine protein excretion (2.6 versus 1.4 g, *p* = 0.02), features associated with worse renal prognosis (6). However, in other lupus nephritis studies, aPL did not correlate with long-term kidney function (7, 8) or even showed a protective effect against renal damage (9).

In addition, several studies demonstrated an association between aPL and a variety of intrarenal vascular lesions in kidney biopsies of patients with lupus nephritis (10). Thrombotic microangiopathy in glomeruli and/or renal arterioles was the most common lesion, characterized by fibrin thrombi without inflammatory cells or immune complexes (11, 12). Several studies from the early 1990s have examined the impact of aPL-associated intrarenal vascular lesions on long-term outcomes of SLE patients (10–14). In 2013, Song et al. reported that thrombotic microangiopathy in patients with lupus nephritis was an independent predictor of poor renal outcome (HR: 2.772, 95% CI 1.009–7.617, *p* = 0.048) (15). Wu et al. showed that thrombotic microangiopathy lesions in lupus nephritis was associated with worse renal prognosis compared to other vascular lesions and suggested that vasculopathy be included in ISN/RPS classification system in order to increase its predictive value for renal outcomes (16).

## Kidney Transplantation and aPL

There is growing evidence that patients with positive aPL and/or APS requiring renal transplantation have increased risk of early graft loss due to post-transplant thrombosis of graft vessels or thrombotic microangiopathy (17, 18). In a large single-center cohort of 1,359 kidney transplantations, the prevalence of aPL was 3%, and LA-positive patients had high rates of allograft aPL-associated vascular lesions and poor transplantation outcomes during the first year after transplantation (18). However, other studies could not confirm an association between aPL and allograft survival after kidney transplantation (19). A very recent study of 446 kidney transplant recipients showed that the risk of GFR decline within the first year post-transplant was elevated in patients with positive aPL, even without thrombotic events prior to transplantation (20).

Perioperative anticoagulation therapy with low molecular weight or unfractionated heparin has been shown to protect from graft failure in patients with positive aPL or APS, However, allograft loss due to thrombosis can develop despite this treatment (17). Anticoagulation treatment can also increase the risk of bleeding complications. Treatment with eculizumab, which blocks the complement cascade at the C5 level, improved post-transplant renal outcomes in case reports of aPL-positive patients with recurrent thrombotic microangiopathy after kidney transplantation (21, 22).

## Renal Artery Thrombosis or Stenosis

Although rare, renal artery thrombosis is a well-recognized clinical manifestation of renal involvement in APS (2). The pathophysiology of renal artery thrombosis implicates either an *in situ* thrombosis or an embolic event in renal artery vasculature. Patients typically present with sudden-onset or uncontrolled systemic hypertension, or the diffuse abdominal or flank pain in the cases of renal infarct (23). The above manifestations in a patient with the diagnosis of APS should raise the suspicion for renal artery thrombosis, and APS should be considered in all cases of well-documented renal artery thrombosis of unknown origin. Renal angiography has the highest diagnostic accuracy, while both contrast-enhanced CT or MRI angiography are less invasive methods with a similar diagnostic performance (24).

Renal artery stenosis without evidence of thrombosis has also been described in the context of APS, representing a significant cause of hypertension in this group of patients. Sangle et al. examined 77 patients with aPL and uncontrolled hypertension, 91 patients attending hypertension clinics, and 92 normotensive healthy controls; renal artery stenosis was diagnosed by magnetic resonance renal angiography in 26, 8, and 3% of each group, respectively (25). A more recent study using ultasonography, for the diagnosis of renal stenosis, showed elevated intrarenal vascular resistance in 14% of APS patients versus none of the aPL carriers (*p* = 0.00007) (26).

Both thrombosis and atherosclerosis have been suggested as major underlying mechanisms for the stenotic lesions (27). In a retrospective study of 23 APS patients, high-intensity anticoagulation (INR ≥ 3) seemed to decrease the rates of renal artery re-stenosis and had a favorable impact on blood pressure control and renal function during their follow-up (28).

## Renal Vein Thrombosis

Either unilateral or bilateral renal vein thrombosis can occur in patients with APS, resulting in acute kidney injury or chronic kidney disease (29, 30). Patients usually present with nephrotic syndrome or less frequently with flank pain and macroscopic hematuria in the acute onset of thrombosis. This diagnosis should be suspected in patients with APS who suddenly develop neprhotic-range proteinuria. Doppler ultrasonography is the examination of choice and may reveal edematous kidney with decreased echogenicity, disruption of parenchymal architecture, and/or thrombus in renal veins.

## APS Nephropathy

Since the early 1990s, thrombotic microangiopathy has been detected in renal biopsies of patients with primary APS (10–13). In addition to thrombotic microangiopathy, Amigo et al. described a number of chronic renal vascular lesions as a part of kidney involvement in APS and in the absence of overt lupus nephritis (31).

**Figure 1:**
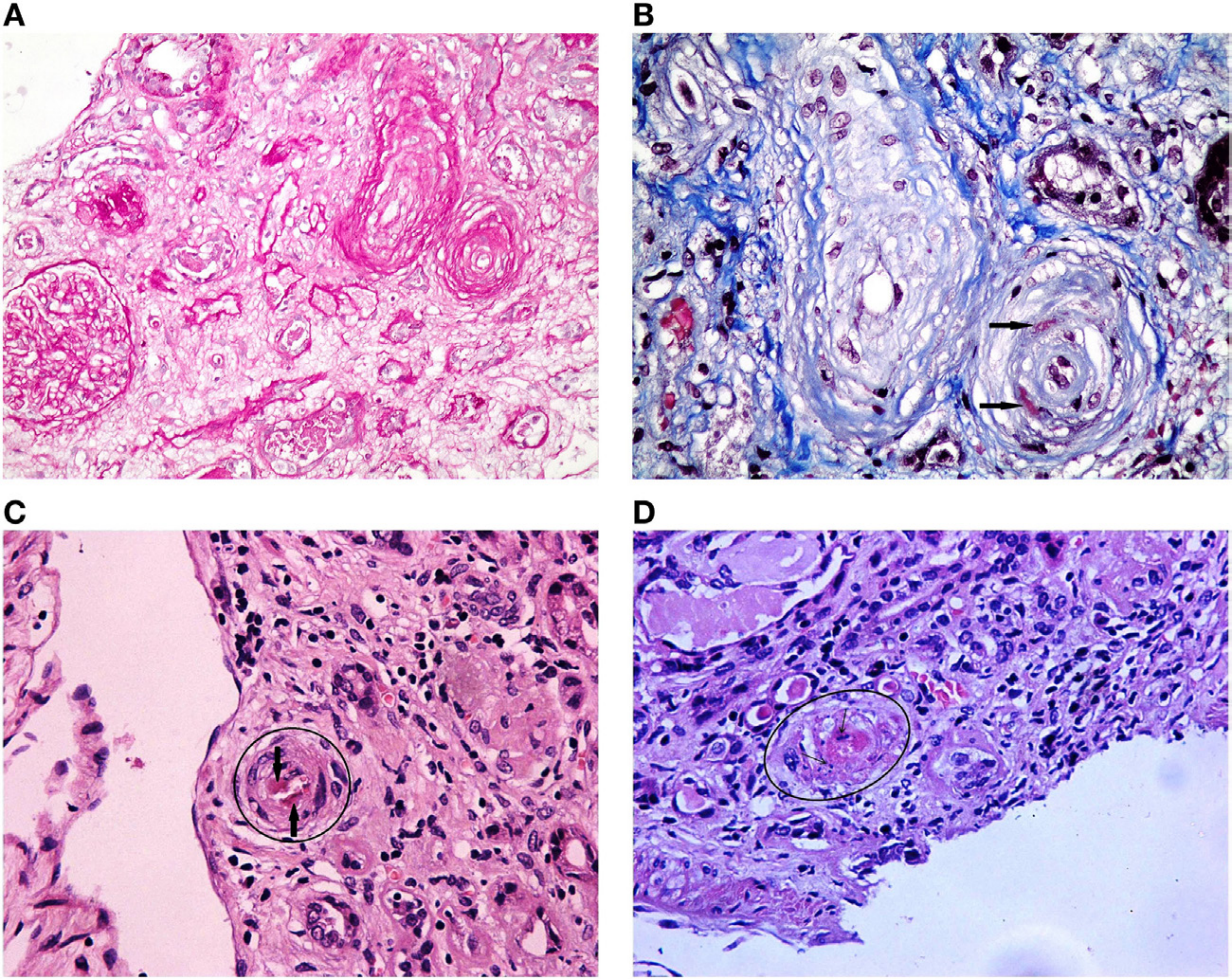
Antiphospholipid syndrome nephropathy histologic lesions. **(A)** Luminal narrowing due to circumferential myointimal thickening of the wall of one arteriole and one interlobular artery. Glomerulus exhibiting ischemic features with wrinkling of the glomerular capillary basement membranes (PAS 200×). **(B)** An interlobular artery and an arteriole showing luminal narrowing due to pale mucoid intimal thickening and myointimal cellular proliferation. Additionally, the arteriole reveals fibrin insudation within the wall (black arrows) (Masson trichrome 400×). **(C)** Arteriole showing luminal thrombus (HE 400×). **(D)** Arteriole showing TMA with platelet-fibrin thrombus occluding the lumen and nuclear debris in the arterial wall (HE 400×).

Antiphospholipid syndrome nephropathy, a renal small-vessel vasculopathy characterized by acute thrombosis and/or chronic arterial and arteriolar lesions, was first defined as a distinct histological and clinical entity in 1999. After examining 16 renal biopsies of primary APS patients, Nochy et al. suggested that at least one of the following lesions should be detected for the diagnosis of APS nephropathy: thrombotic microangiopathy (acute lesion), interlobular fibrous intimal hyperplasia, arterial and arteriolar recanalizing thrombi, fibrous arterial occlusion, and focal cortical atrophy (32) (Figure 1). The same French group later observed the same histological lesions in patients with SLE-associated APS, over and above lupus nephritis lesions (33). APS nephropathy has been associated with LA, arterial thrombosis, and fetal loss. It was also associated with an higher risk of hypertension, elevated serum creatinine levels, and kidney interstitial fibrosis, all recognized as predictors of worse renal outcomes.

Tektonidou et al. showed that in lupus nephritis biopsy samples, APS nephropathy lesions were much more prevalent in aPL-positive patients (39.5% versus only 4.3% of those with negative aPL) (34). Furthermore, APS nephropathy was found in two-thirds of those meeting APS criteria among those aPL-positive patients with SLE. A strong association with APS nephropathy was also noted in patients with arterial thrombosis and livedo reticularis. APS nephropathy was characterized by a higher frequency of hypertension and elevated creatinine levels on biopsy, but did not predict the risk of decline in kidney function, end-stage renal disease or death at the end of follow-up. The rate of APS-related clinical manifestations, such as arterial thrombosis, was higher in SLE patients with versus without APS nephropathy during a long-term follow-up.

Some years later, Tektonidou et al. examined three different APS groups for acute and chronic APS nephropathy lesions: primary APS, SLE-associated APS, and for the first time, catastrophic APS (35). Thrombotic microangiopathy, the acute lesion, was prominent in catastrophic APS while the prevalence of chronic lesions was similar among all APS groups. In all three APS groups, hypertension, proteinuria (mild to nephrotic syndrome), microscopic hematuria, and renal insufficiency (usually mild) were the main clinical features of APS nephropathy.

Further studies confirmed the above findings. However, the impact of APS nephropathy on long-term renal outcomes varied among different studies. In a single cohort from Thailand, APS nephropathy lesions were present in 34% of 150 patients with biopsy-proven lupus nephritis. APS nephropathy was correlated with indices of disease activity and chronicityhypertension, renal failure, severe proteinuria, class III and IV histology, and end-stage renal disease (36). In another study, APS nephropathy was present in 10% of kidney biopsy specimens from 162 Mexican patients with lupus nephritis and was associated with anticardiolipin antibodies and elevated rates of rapidly progressive glomerulonephritis, nephrotic syndrome, and death during follow-up (37). In a Spanish cohort of 77 SLE patients with biopsy-proven renal involvement, a strong correlation was found between APS nephropathy and aPL (*p* = 0.003), especially the combination of LA and IgG anticardiolipin antibodies (OR: 3.61, *p* = 0.002). The levels of serum creatinine were higher in APS nephropathy patients (*p* = 0.038), however, no significant difference in complete or partial remission, not response, and chronic renal damage was observed between the two groups (38).

In 2011, all published studies examining the association between aPL and APS nephropathy were identified and graded in the context of a “Task Force on Non-criteria APS Manifestations” (39). The task force group reported a higher frequency of APS nephropathy in patients with positive aPL (*p* < 0.001) compared to those without aPL (Evidence Level II) and in primary APS compared to SLE-APS, and to SLE with positive aPL but without APS. The specificity, positive predictive value, and negative predictive value of APS nephropathy for the detection of APS were 96, 85 and 87, respectively. Some years later, another task force group evaluated the relevance of non-criteria clinical manifestations of APS according to the GRADE system to support their inclusion in the APS classification criteria (40). The overall quality of evidence was “very low” or “low” for most of non-criteria manifestations, but “moderate” for APS nephropathy.

Renal pathologists should be aware of this histological entity, and clinicians should include APS in the differential diagnosis of small-vessel nephropathy. Because thrombotic microangiopathy is a non-specific lesion, other conditions associated with its presence should be ruled out such as thrombotic thrombocytopenic purpura, atypical hemolytic uremic syndrome, HELLP syndrome, malignant hypertension, systemic sclerosis, preeclampsia or eclampsia, and medications (cyclosporine, chemotherapy).

## Glomerular Lesions in Primary APS

Fakhuri et al. found APS nephropathy lesions in 20 of 29 biopsies of primary APS patients, and they reported a variety of glomerular lesions such as membranous nephropathy, minimal change disease/focal segmental glomerulosclerosis, mesangial C3 nephropathy, and pauci-immune crescentic glomerulonephritis (41). Additional case reports described histologic lesions of proliferative glomerulonephritis in primary APS patients with biopsy-proven renal involvement, with no evidence of thrombotic microangiopathy or any other APS-related renal vascular lesions (42, 43).

An Italian multicenter cohort study of 160 primary APS patients examined the kidney biopsy findings of 10 patients with evidence of renal involvement. Four patients had findings consistent with APS nephropathy, four had membranous, and two had proliferative glomerulonephritis. Patients with renal involvement were older (*p* = 0.0269), had positive LA test (*p* = 0.0068), and low complement levels (*p* < 0.05) (44).

## Pathophysiology of APS Nephropathy

The significant association between the presence of APS nephropathy and aPL suggests a pathogenetic role of aPL in the development of this nephropathy. However, it is unknown if additional disease-related factors contribute to the pathogenesis of APS nephropathy. It is also unclear whether this is a purely thrombotic or inflammatory process. Complement cascade activation has been recognized as an important mechanism for aPL-mediated thrombosis in murine models (45, 46). Experimental studies also showed that absence of complement regulatory proteins on glomerular cells is associated with thrombotic microangiopathy (47). In clinical studies, C4d is a common finding in thrombotic microngiopathy (48). C4d staining and microthrombi were found to co-exist in biopsy samples of patients with SLE and positive aPL (49, 50).

In addition, complement-mediated tissue factor seems to be involved in the pathogenesis of thrombotic microangiopathy in APS (51). Seshan et al. showed that mouse aPL and human aPL of IgG isotype can induce glomerular histologic lesions characteristic of thrombotic microangiopathy in mice. They also found an increased deposition of fibrin, tissue factor, and C3 in glomeruli of mice treated with mouse and human aPL supporting their role in thrombotic microngiopathy pathogenesis (52).

Antiphospholipid syndrome nephropathy is also characterized by a number of chronic lesions with fibrous intimal hyperplasia being the most common. A recent study in patients with APS showed activation of the mTORC pathway in the renovascular endothelium, leading to intimal hyperplasia. Vascular activation of mTORC was also demonstrated in autopsy specimens of a catastrophic APS case series (53).

## Treatment of APS Nephropathy

Currently, a consensus on the treatment of APS nephropathy is lacking. Patients with APS nephropathy histologic lesions who fulfill the clinical and laboratory criteria for APS (1) should receive the standard anticoagulant treatment for APS. However, whether anticoagulation or other treatment is indicated in patients with APS nephropathy lesions in the absence of definite APS criteria is not well established. In patients with co-existent lupus nephritis, the use of hydroxychloroquine and immunosuppressive treatment is recommended (54). Since hypertension and proteinuria are predominant manifestations of APS nephropathy, the standard of care includes inhibitors of the angiotensin system (55).

The role of anticoagulation in renal prognosis has not been well examined due to the limited number of patients on anticoagulation in the previous studies of APS nephropathy. Prospective studies are lacking. A successful use of anticoagulants was reported in some case reports or case series but with no or short follow-up data (56). The use of novel oral anticoagulant medications has not been examined in patients with APS nephropathy. Other treatment approaches may include the use of heparin based on its effect on the classical complement activation pathway inhibition or the use of intravenous immunoglobulin and/or plasma exchange given their efficacy on severe/refractory cases and catastrophic APS (56, 57). Targeted therapies may also be considered such as B-cell directed therapies, complement inhibition, tissue factor inhibition, or mTOR pathway inhibition, but large prospective multicenter studies are needed to address their role.

Experimental APS murine models have shown that the use of BAFF blocking agents delays the development of disease and prolongs survival (58). In humans, case reports showed a successful use of anti-CD20 treatment in patients with APS nephropathy and/or other non-criteria manifestations for APS (severe thrombocytopenia, hemolytic anemia, and skin ulcers) (59). In a 12-month, unblinded study evaluating the potential usefulness of rituximab for non-criteria manifestations of APS, two patients with APS nephropathy had partial responses with two doses of 1,000 mg rituximab on days 1 and 15 (60). Belimumab, a BAFF antagonist, has been used in two cases with primary APS, one with recurrent alveolar hemorrhage and one with recurrent skin ulcers. Both patients had clinical improvement and were able to discontinue corticosteroids (61).

Regarding T-cell-directed therapies, an experimental study of CTLA4-Ig in the NZW/BXSB mice that develop APS with small coronary thrombosis, showed promise in preventing the development of myocardial infarcts (62). There is no available evidence about the use of co-stimulation blockade Abatacept (CTLA4-Ig), in patients with APS.

Regarding complement inhibition in APS, animal studies demonstrated the ability of C5 inhibitors to prevent blood clots in animals receiving intravascular infusion of antibodies to β2GPI (50). Seshan et al. showed that genetic deletion of C5aR prevents thrombotic microangiopathy and renal failure in aPL (FB1)-treated mice (52). Eculizumab, a recombinant humanized monoclonal antibody that binds to C5 inhibiting its cleavage to C5a and C5b, has been successfully used in “thrombotic microangiopathy” group of disorders (characterized by thrombocytopenia and microangiopathic hemolytic anemia), including paroxysmal nocturnal hemoglobinuria, hemolytic uremic syndrome, and catastrophic APS. Case reports have described its effect in refractory catastrophic APS cases (63), post kidney transplant thrombotic microangiopathy (22), and patients with lupus nephritis and thrombotic microangiopathy (64).

The role of statin use in APS has also been extensively discussed. Experimental models showed that low tissue factor expression prevents glomerular injury in mouse aPL- and human aPL-treated mice. Pravastatin, which down-regulates tissue factor, prevents glomerular injury in both mouse aPL- and human aPL-treated mice (52).

Inhibition of mTOR pathway in kidney transplant recipients with APS nephropathy decreased vascular proliferation on renal biopsy and prevented vascular lesion recurrence. Canaud et al. showed that among the 10 patients who received mTOR pathway inhibition treatment, 7 patients (70%) had a functioning allograft 10 years after transplantation versus 3 (11%) of 27 untreated patients, and the effect of treatment was independent of anticoagulation treatment (53).

Two recent studies showed a type I interferon signature in primary APS (65, 66). The first study showed an impaired ability of endothelial progenitors from patients with APS to differentiate into endothelial cells, reversed by a type I IFN receptor-neutralizing antibody (65). The above findings could support a novel therapeutic approach, that of anti-interferon antibodies, to reverse vascular damage in APS.

## Conclusion

Manifestations associated with renal vasculature involvement in the presence of persistently positive aPL and/or APS include renal artery or vein thrombosis, thrombotic microangiopathy lesions in lupus nephritis biopsies, allograft thrombosis after kidney transplantation, and a small-vessel nephropathy characterized as APS nephropathy with variable outcomes. Better understanding of the pathogenetic mechanisms of APS nephropathy may lead to more precise and targeted treatments.

## Author Contributions

MT drafted the manuscript.

## Conflict of Interest Statement

The author declares that the research was conducted in the absence of any commercial or financial relationships that could be construed as a potential conflict of interest.
